# Post-Hospitalisation COVID-19 Rehabilitation (PHOSP-R): a randomised controlled trial of exercise-based rehabilitation

**DOI:** 10.1183/13993003.02152-2024

**Published:** 2025-05-22

**Authors:** Enya Daynes, Rachael A. Evans, Neil J. Greening, Nicolette C. Bishop, Thomas Yates, Daniel Lozano-Rojas, Kimon Ntotsis, Matthew Richardson, Molly M. Baldwin, Malik Hamrouni, Emily Hume, Hamish McAuley, George Mills, Dimitrios Megaritis, Matthew Roberts, Charlotte E. Bolton, James D. Chalmers, Trudie Chalder, Annemarie B. Docherty, Omer Elneima, Ewen M. Harrison, Victoria C. Harris, Ling P. Ho, Alex Horsley, Linzy Houchen-Wolloff, Olivia C. Leavy, Michael Marks, Krishna Poinasamy, Jennifer K. Quint, Betty Raman, Ruth M. Saunders, Aarti Shikotra, Amisha Singapuri, Marco Sereno, Sarah Terry, Louise V. Wain, William D-C. Man, Carlos Echevarria, Ioannis Vogiatzis, Christopher Brightling, Sally J. Singh

**Affiliations:** 1 The Institute for Lung Health, NIHR Leicester Biomedical Research Centre, University Hospitals of Leicester, Leicester, UK; 2 Department of Respiratory Sciences, University of Leicester, Leicester, UK; 3 National Centre for Sport and Exercise Medicine, School of Sport, Exercise and Health Sciences, Loughborough University, Loughborough, UK; 4 NIHR Leicester Biomedical Research Centre – Diabetes, Leicester, UK; 5 Diabetes Research Centre, College of Life Sciences, University of Leicester, Leicester, UK; 6 Department of Sport, Exercise and Rehabilitation, Faculty of Health and Life Sciences, Northumbria University, Newcastle upon Tyne, UK; 7 Centre for Respiratory Research, Translational Medical Sciences, School of Medicine, University of Nottingham, Nottingham, UK; 8 NIHR Nottingham Biomedical Research Centre, Nottingham, UK; 9 University of Dundee, Ninewells Hospital and Medical School, Dundee, UK; 10 Institute of Psychiatry, Psychology and Neuroscience, King's College London, London, UK; 11 Centre for Medical Informatics, The Usher Institute, University of Edinburgh, Edinburgh, UK; 12 MRC Human Immunology Unit, University of Oxford, Oxford, UK; 13 NIHR Oxford Biomedical Research Centre, Oxford, UK; 14 Manchester University NHS Foundation Trust, Manchester, UK; 15 Department of Population Health Sciences, University of Leicester, Leicester, UK; 16 Department of Clinical Research, London School of Hygiene and Tropical Medicine, London, UK; 17 Hospital for Tropical Diseases, University College London Hospital, London, UK; 18 Asthma and Lung UK, London, UK; 19 School of Public Health, Imperial College London, London, UK; 20 Radcliffe Department of Medicine, University of Oxford, Oxford, UK; 21 Oxford University Hospitals NHS Foundation Trust, Oxford, UK; 22 Harefield Respiratory Research Group, Royal Brompton and Harefield Hospitals, Guy's and St Thomas’ NHS Foundation Trust, London, UK; 23 National Heart and Lung Institute, Imperial College, London, UK; 24 King's Centre for Lung Health, Faculty of Life Sciences and Medicine, King's College London, London, UK; 25 The Newcastle upon Tyne Hospitals NHS Foundation Trust, Translational and Clinical Research Institute, Newcastle University, Newcastle upon Tyne, UK

## Abstract

**Objective:**

Post-COVID syndrome involves prolonged symptoms with multisystem and functional impairment lasting ≥12 weeks after acute coronavirus disease 2019 (COVID-19). We aimed to determine the efficacy of exercise-based rehabilitation interventions, either face-to-face or remote, compared to usual care in individuals experiencing post-COVID syndrome following a hospitalisation with acute COVID-19.

**Design:**

This single-blind randomised controlled trial compared two exercise-based rehabilitation interventions (face-to-face or remote) to usual care in participants with post-COVID syndrome following a hospitalisation. The interventions were either a face-to-face or remote 8-week programme of individually prescribed exercise and education. The primary outcome was the change in Incremental Shuttle Walking Test (ISWT) following 8 weeks of intervention (either face-to-face or remote) compared to usual care. Other secondary outcomes were measured including health-related quality of life (HRQoL), and exploratory outcomes included lymphocyte immunotyping.

**Results:**

181 participants (55% male, mean±sd age 59±12 years, length of hospital stay 12±19 days) were randomised. There was an improvement in the ISWT distance following face-to-face rehabilitation (mean 52 m, 95% CI 19–85 m; p=0.002) and remote rehabilitation (mean 34 m, 95% CI 1–66 m; p=0.047) compared to usual care alone. There were no differences between groups for HRQoL self-reported symptoms. Analysis of immune markers revealed significant increases in naïve and memory CD8^+^ T-cells following face-to-face rehabilitation *versus* usual care alone (p<0.001, n=31).

**Conclusion:**

Exercise-based rehabilitation improved short-term exercise capacity in post-COVID syndrome following an acute hospitalisation and showed potential for beneficial immunomodulatory effects.

## Shareable abstract

**Rehabilitation improved short-term exercise capacity in long COVID replicated by real-world evidence and had beneficial immunomodulatory effects** https://bit.ly/42l0wzk

## Graphical abstract

**Figure:**
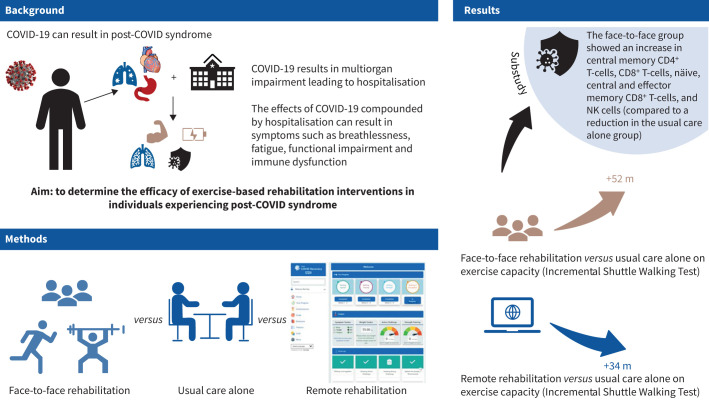
In this study individuals with post-COVID syndrome improved exercise capacity following face-to-face rehabilitation or digital rehabilitation compared to usual care alone.

## Introduction

Coronavirus disease 2019 (COVID-19) is a complex, multisystem condition caused by infection with severe acute respiratory syndrome coronavirus 2 (SARS-CoV-2). COVID-19 resulted in >1 million hospital admissions in the United Kingdom, which was ∼5–10% of all individuals infected with COVID-19. Symptoms lasting >12 weeks are termed “post-COVID syndrome” or “long COVID” [1, 2]. The World Health Organization (WHO) estimates that 10–20% of people infected with COVID will experience post-COVID syndrome; however, true estimates are unknown [3]. Up to 70% of individuals in the Post-Hospitalisation (PHOSP)-COVID study reported an incomplete recovery at 1 year following hospitalisation, although this continues to improve, as reported by the Global Burden of Disease, with approximately one-third of those infected self-reporting symptoms of post-COVID syndrome at 3 months and 15.1% at 12 months [4, 5]. Individuals with post-COVID syndrome experience a range of symptoms (*e.g.* breathlessness, fatigue) leading to functional impairment, reduced exercise capacity and difficulty performing activities [4]. The exact mechanisms of these impairments are not entirely understood, but it is likely to be an interaction of ongoing pathology including acute treatment complications, immune system dysregulation and ongoing inflammation, compounded by the impacts of a hospitalisation [6–8].

In chronic respiratory diseases, comprehensive rehabilitation programmes comprising individually prescribed and progressed exercise and education significantly improve symptoms (*e.g.* breathlessness and fatigue), exercise intolerance and health-related quality of life (HRQoL) [6]. These benefits have been demonstrated in other post-hospitalisation cohorts [9]. Given the overlap between some symptoms of post-COVID syndrome and those with other chronic diseases, it is plausible that similar rehabilitation programmes may convey comparable benefits [6]. This is supported by the WHO recommendation to consider rehabilitation for those with post-COVID syndrome, and specifically to provide a programme of education and support for self-management of breathlessness, resumption of activities and a gradual increase of exercise based on symptoms [10].

Previous studies have demonstrated maladaptation in the immune system following COVID-19 with decreases in T-cell and natural killer (NK) immune cell populations and a reduced frequency and number of naïve CD4 and CD8 T-cells in those with severe symptoms compared to healthy controls [11]. Evidence in other respiratory diseases has highlighted that exercise rehabilitation can improve CD4^+^ T-cells and reduce hospitalisations. It is therefore plausible that exercise-based rehabilitation may impact the immune system in post-COVID syndrome [12].

Several systematic reviews demonstrated improvements in symptoms following rehabilitation interventions, although the evidence is heterogeneous, with a high risk of bias, and consists predominantly of uncontrolled trials and poorly defined interventions [13–15]. Early evidence supports this hypothesis, demonstrating increased exercise tolerance and improved respiratory symptoms, fatigue and cognition in individuals with post-COVID syndrome [16]. Remote programmes performed synchronously as a group (REGAIN trial) have been shown to improve quality of life for individuals with post-COVID syndrome [17], but did not include prescribed exercise training following exercise testing.

Despite the promise of existing evidence, many patients describe symptoms that present a challenge and could impact engagement [18–20]. Therefore, there is a need to investigate exercise-based rehabilitation programmes by different modes of delivery: supervised, face-to-face rehabilitation programmes for those who can attend and where a digital solution is not acceptable; and asynchronous remotely monitored digital methods that are flexible to cater for the needs of individuals with post-COVID syndrome.

In this randomised controlled trial (RCT), we hypothesised that face-to-face and remote interventions added to usual care would improve exercise capacity compared to usual care alone in individuals with post-COVID syndrome, following a hospitalisation. A subgroup analysis explored the response of immune biomarkers to face-to-face rehabilitation.

## Methods

This study consisted of a single-blind, three-arm RCT conducted at the University of Leicester and Northumbria University. The trial was approved by Yorkshire and the Humber – Leeds West research ethics committee (reference number: 20/YH/0225) and registered on the ISRCTN trial registry (identifier numbers ISRCTN10980107/ISRCTN13293865). Full details of the methodology are available and described in detail [21].

### Participants

Participants were eligible if they were adults aged ≥18 years; admitted to hospital during a confirmed acute episode of COVID-19 (PCR-positive or clinician diagnosed); and had ongoing symptoms lasting >12 weeks resulting in self-described functional impairment. Participants had a clinician-determined diagnosis of post-COVID syndrome in the specialist COVID clinic prior to referral.

Individuals were excluded if they had any contraindication to exercise [22]; experienced symptoms indicative of another medical condition that required further investigation/management (*i.e.* clinical diagnosis or self-reported severe post-exertional malaise (PEM)/post-exertional symptom exacerbation (PESE) rendering the individual bedbound, or postural orthostatic tachycardia syndrome); unstable comorbidities; or completion of a rehabilitation programme in the preceding 6 months.

### Randomisation and masking

Block randomisation (block size 6) was performed on www.sealedenvelope.com with allocation concealment by unblinded members of the study team who arranged the intervention. A randomisation log was maintained by the unblinded study team. A participant's ability to undertake each intervention was determined through discussion with a healthcare professional (*e.g.* low digital literacy preventing remote rehabilitation). Participants able to access any intervention were randomised in a 1:1:1 ratio. Those unable to access one of the interventions (either face-to-face or remote) were randomised in a 2:1 ratio in favour of the remaining intervention. This was based on ability to access, rather than preference. Outcome assessors and data analysts were blinded to intervention allocation. There were no incidences of unblinding throughout the trial.

### Procedures

The interventions have been described fully elsewhere [21]. The intervention phase was 8 weeks in duration. The exercise component was individually prescribed using the Incremental Shuttle Walking Test (ISWT) which calculates a predicted maximal oxygen uptake, and intensity is prescribed aiming for moderate–high intensity training where able (∼80–85% of maximum) and tailored in response to symptoms. Usual care was offered to all three groups.

#### Face-to-face rehabilitation

The programme comprised twice weekly face-to-face sessions (∼90–120 min per session) involving symptom-titrated exercise training (aerobic (walking and cycling) and resistance); a package of education (16 topics in total; supplementary table S1); self-management strategies including individualised pacing, prioritising and planning advice, 1:1 management and symptom advice and vocational advice where relevant. This was delivered by a multidisciplinary team including physiotherapists, occupational therapists, nurses, exercise physiologists and support assistants. Educational sessions followed an interactive discussion-based approach (rather than didactic lectures). Exercise was individually tailored following a comprehensive assessment and supplemented by an individualised home exercise programme recorded in a symptom and activity diary. The home exercise programme consisted of an additional three sessions, typically two aerobic only and one both strength and aerobic, as indicated. All symptoms in response to exercise were monitored by a healthcare professional at sessions and the programme was individually tailored accordingly. Adjustments to frequency or intensity, or exercise adaptations (for example changing exercise components due to pain) were made. This was a group programme and therefore participants also received informal peer support.

#### Remote rehabilitation

The intervention used the YourCOVIDRecovery (now hosted within https://www.i-impact.co.uk/) platform which was a password protected site comprised of four phases, allocating two weeks per phase. This was a remotely monitored programme where participants completed symptom-titrated exercise training (aerobic (walking) and resistance) and self-directed symptom management advice supported by a healthcare professional. Equipment was not provided for home use. Participants were supported by a healthcare professional through fortnightly phone calls and the website messaging service as required; this included a 1:1 discussion to monitor progression and support implementation of self-management strategies, offer individual symptom advice and vocational advice where relevant. Symptoms and exercises were monitored on the platform and the education was tailored accordingly. High scoring/worsening of symptoms triggered a notification to a healthcare professional and support/follow-up was offered as required, either through direct messaging or telephone contact.

#### Usual care

Usual care was offered to all groups and ensured that participants could access any treatment that was offered in the management of their post-COVID syndrome. All participants were seen in a specialist outpatient COVID clinic hosted within secondary care, led by a multidisciplinary team of consultants (respiratory, cardiology, neurology, diabetologist, renal and general practitioner), nurses and physiotherapists. Participants had their care optimised prior to enrolment in the trial through the COVID clinic; the frequency of appointments was tailored to the individual's needs. When this trial was conducted, treatment was not standardised, but was offered based on an individual and thorough assessment; this could include, but was not limited to, psychological interventions, medical treatment, symptom self-management or breathing pattern retraining. As rehabilitation was not considered usual care at trial setup, participants were excluded from engaging in exercised-based rehabilitation during the trial period. The control group for this study was usual care alone.

### Outcome measures

#### Primary outcome

The primary outcome was the change in the ISWT [23] reported in distance walked (metres) pre- and post-intervention. The comparison focused on one intervention (either face-to-face rehabilitation or remote rehabilitation) compared to usual care alone from pre- to post-intervention. The ISWT was completed in line with European Respiratory Society/American Thoracic Society technical standards, on a 10-m track, and included a familiarisation test [23].

#### Secondary outcomes

Physical measurements included the Short Physical Performance Battery (SPPB), handgrip strength and quadriceps strength using quadriceps maximum isometric voluntary contraction (QMVC). Symptoms and HRQoL were assessed using self-reported questionnaires as, follows: EuroQol five-dimension five-level questionnaire (EQ-5D), Patient Health Questionnaire (PHQ)-9, the Generalised Anxiety Disorder seven-item scale (GAD-7), Dyspnoea-12, the Functional Assessment of Chronic Illness Therapy Fatigue Scale (FACIT-FS), the DePaul Symptom Questionnaire, and the Brief Pain Inventory. Cognition was assessed using the Montreal Cognitive Assessment (MoCA). These measures were completed pre- and post-intervention.

The modified Medical Research Council dyspnoea scale, SARC-F (strength, assistance in walking, rise from a chair, climb stairs, falls), General Practice Physical Activity Questionnaire and Nijmegen Questionnaire were used to describe participant characteristics at baseline. Participants were categorised by WHO Severity Index as 1) nonsevere requiring hospitalisation, but no ventilatory support; 2) severe requiring oxygen therapy (including high-flow); or 3) critical requiring mechanical ventilation (invasive or noninvasive).

#### Exploratory outcomes

Venous immune outcomes were collected on a subset of participants in the face-to-face rehabilitation and usual care group (n=40), including T-cells (naïve, central memory, effector memory and terminally differentiated effector memory), and NK cells (further details in the supplementary material, and supplementary figure S1) pre- and post-intervention. Flow cytometry was performed to analyse immune cell subsets using fluorescently conjugated antibodies.

A subgroup of participants received optional muscle biopsies (as described in the trial protocol); however, the results are not available at present.

### Statistical analysis

The sample size was based on a mean difference between groups of 50 m in the primary outcome (ISWT), with a standard deviation of 72 m taken from previous cohort studies [16]. The study was powered at 90% with a type I error of two-sided α=0.05, and required 44 participants per group and 132 participants in total. The sample size was inflated by 20% to account for attrition to give a final target sample size of 159 participants [21].

Statistical analysis was performed in R version 4.2.0 and the analysis compared the interventions separately (face-to-face or remote) to usual care alone. The primary analysis utilised a generalised linear mixed models (GLMM) with an intention-to-treat approach. Assumptions were assessed (supplementary material). The GLMM predicts the ISWT accounting for random variability of participants to be excluded from the correlations between covariates and the model's output variable. The GLMM considers the baseline of participants that did not complete the post-intervention ISWT. The GLMM compared changes from baseline following 8 weeks of either face-to-face rehabilitation to usual care or remote rehabilitation to usual care and within group changes were calculated by the estimation of marginal mean differences. Independent variables included the interaction between time point and treatment group (face-to-face *versus* usual care and remote *versus* usual care), with age, sex, body mass index (BMI), time since hospitalisation, number of comorbidities, WHO Severity Index, and recruiting site included as fixed independent variables in the model. An interaction term, time point×group, to measure a difference between treatments and a random intercept per individual (random effect) was included in the model. Further information on statistical analysis, including per-protocol analysis and immune subgroup analysis can be found in the supplementary material (supplementary figures S2–S4, supplementary table S2).

## Results

**FIGURE 1:**
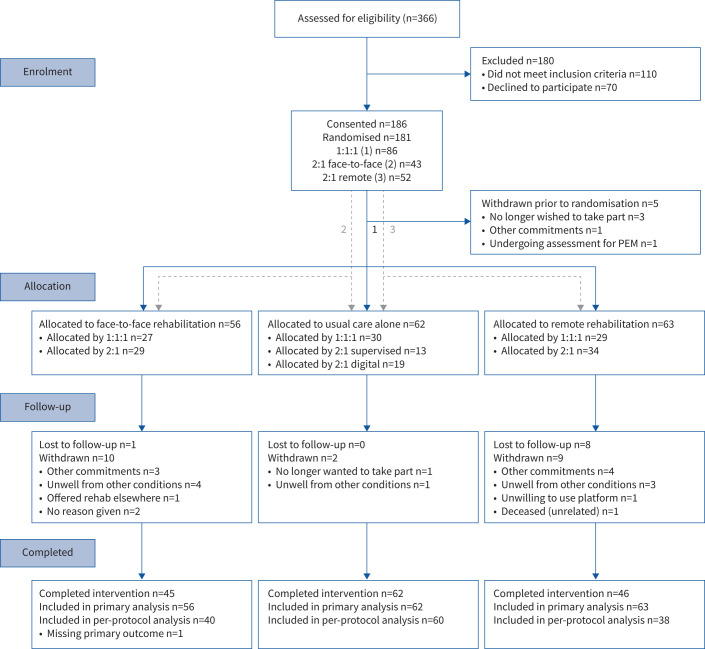
Consort diagram for PHOSP-R study. Randomisation procedure indicated as 1) following a 1:1:1 ratio, 2) following a 2:1 ratio in favour of face-to-face rehabilitation and 3) following a 2:1 ratio in favour of digital rehabilitation. PEM: post-exertional malaise.

181 participants were randomised (mean±sd age 59±12 years, n=99 (55%) male, n=142 (78%) white British, n=54 (30%) critical WHO Severity Index, requiring ventilatory support, at the time of admission) between March 2022 and May 2023. The median (interquartile range (IQR)) length of stay was 6 (1–12) days and mean±sd time since initial infection was 545±211 days. 123 (68%) participants were recruited at the University of Leicester and 58 (32%) were recruited from Northumbria University, Newcastle. 86 (48%) participants were able to attend either face-to-face or remote interventions; 53 (29%) were unable to attend a face-to-face programme, primarily due to other commitments; and 42 (23%) were unable to access a remote intervention, primarily due to limited access to digital technologies. 149 (82%) participants completed the trial, providing primary outcome data at follow-up (figure 1). Full baseline characteristics are shown in table 1.

**TABLE 1:** Baseline characteristics of study participants according to the randomised groups (regardless of randomisation procedure)

	Face-to-face	Remote	Usual care	Total
**Participants**	56	63	62	181
**Age (years)**	61±13	55±11	62±11	59±12
**Male**	31 (55)	29 (46)	39 (63)	99 (55)
**Ethnicity**				
White	44 (79)	50 (79)	48 (77)	142 (78)
Asian	9 (16)	12 (19)	10 (16)	31 (17)
Other	3 (5)	1 (2)	4 (6)	8 (4)
**BMI (kg·m^−2^)**	32.0±3.6	31.5±3.5	31.3±3.2	31.6±3.4
**Index of Multiple Deprivation**	6 (3–9)	5 (5–8)	6 (4–8)	6 (3–8)
**Comorbidities**				
0 comorbidities	11 (20)	20 (32)	11 (18)	42 (23)
1 comorbidity	11 (20)	9 (14)	15 (24)	35 (19)
≥2 comorbidities	34 (61)	34 (54)	36 (58)	104 (57)
**WHO severity classification during hospital admission**				
Nonsevere	14 (25)	22 (35)	27 (44)	63 (35)
Severe	23 (41)	17 (27)	20 (32)	60 (33)
Critical	19 (34)	22 (35)	13 (21)	54 (30)
**Length of hospital stay (days)**	7 (4–14)	5 (1–13)	4 (0–10)	6 (1–12)
**Time since hospitalisation (days)**	578±176	542±219	519±232	545±211
**MRC dyspnoea scale**	3 (2–4)	2 (2–3)	3 (2–3)	3 (2–4)
**SARC-F score**	2 (1–4)	2 (0–4)	2 (1–4)	2 (1–4)
**Nijmegen Questionnaire**	20±12	23±13	21±12	21±13
**General Practice Physical Activity Questionnaire**				
Inactive	31 (55)	33 (53)	35 (56)	99 (55)
Moderately inactive	14 (25)	6 (10)	4 (6)	24 (13)
Moderately active	5 (9)	12 (19)	10 (16)	27 (15)
Active	6 (11)	12 (19)	11 (18)	29 (16)

Data are presented as n, mean±sd, n (%) or median (interquartile range). Measures are at first study visit unless indicated otherwise. BMI: body mass index; WHO: World Health Organization; MRC: Medical Research Council; SARC-F: strength, assistance in walking, rise from a chair, climb stairs, falls.

**FIGURE 2:**
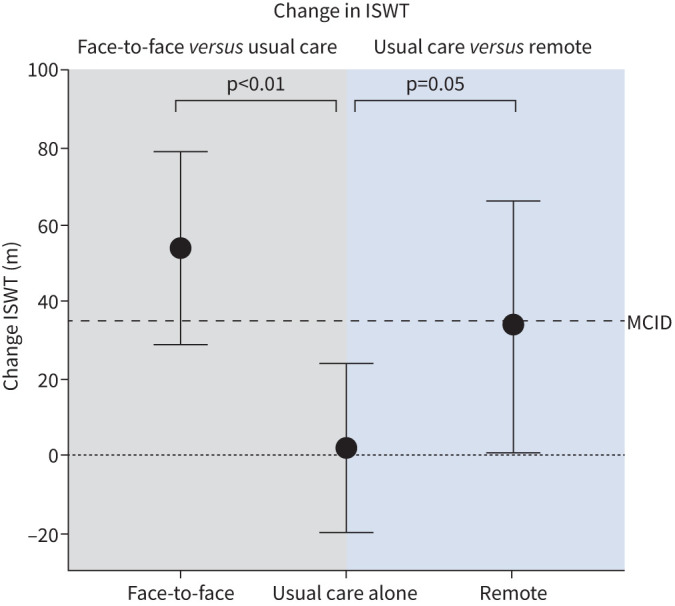
Mean (95% confidence interval) change from baseline for face-to-face, usual-care and remote groups. Comparisons are made between face-to-face and usual care, and remote and usual care. ISWT: Incremental Shuttle Walking Test; MCID: minimal clinically important difference.

The primary adjusted analysis demonstrated a statistically significant improvement in ISWT between the face-to-face rehabilitation and usual-care group with a mean (95% CI) difference of 52 (19–85) m in favour of the intervention (p=0.002) (figure 2). The face-to-face rehabilitation group improved from 285 (219–351) m to 312 (244–380) m (p<0.001). The unadjusted analysis demonstrated a statistically significant difference of 55 (19–92) m in favour of the intervention (p<0.001).

The primary adjusted analysis demonstrated a statistically significant difference between the remote rehabilitation and usual-care group with a mean (95% CI) difference of 34 (1–66) m in favour of the intervention (p=0.047) (figure 2). The remote rehabilitation group improved from 353 (296–411) m to 388 (334–442) m (p<0.01). The unadjusted analysis demonstrated a statistically significant difference with a difference of 49 (13–86) m in favour of the intervention (p<0.001).

140 out of 181 participants were included in the per-protocol analysis: 40 out of 56 (71%) face-to-face, 38 out of 62 (61%) remote and 60 out of 62 (98%) usual-care participants completed 75% of the intervention and the follow-up measures. The difference between the face-to-face rehabilitation and usual-care group was 66 (32–100) m in favour of the intervention (p<0.001). The difference between the remote rehabilitation and usual-care group was 42 (7–78) m in favour of the intervention (p=0.021) (supplementary table S3). Participants achieving >35 m improvement numbered 25 (56%), 22 (50%) and 11 (19%) for the face-to-face, remote and usual-care groups, respectively.

Secondary outcomes are detailed in table 2. There were clinical improvements in the SPPB compared to usual care for both the face-to-face and remote interventions (median (IQR) 1.2 (−0.01–2.38) and 1.5 (0.27–2.66), respectively). There were clinical improvements in the 4-m gait speed test compared to usual care for both the face-to-face and remote interventions (mean (95% CI) 0.12 (−0.01–0.21) m·s^−1^ and 1.5 (−0.05–0.14) m·s^−1^, respectively). There were clinical important improvements in the QMVC compared to usual care for the face-to-face and remote interventions (3.33 (−0.55–7.10) kg and 3.35 (0.43–7.10) kg, respectively). There were clinically important improvements in handgrip strength in the face-to-face rehabilitation programme compared to usual care (2.06 (0.07–4.18) kg). There were no differences between groups for HRQoL or self-reported symptoms measured by the EQ-5D, Dyspnoea-12, PHQ-9, GAD-7, FACIT-FS, MoCA and the DePaul Symptom Questionnaire. There were clinically important within-group improvements in fatigue for face-to-face rehabilitation (table 2). The per-protocol analysis demonstrated similar changes (supplementary tables S3 and S4).

**TABLE 2:** Secondary outcomes for face-to-face rehabilitation *versus* usual care, and remote rehabilitation *versus* usual care

	Face-to-face			Remote			Usual care			Face-to-face *versus* usual care	Remote *versus* usual care
	Pre-trial	Post-trial	Change	Pre-trial	Post-trial	Change	Pre-trial	Post-trial	Change	(difference)	(difference)
**Participants**	56			62			63				
**SPPB**^#^ **(median (IQR))**	9 (7–11)	10 (8–12)	1.5 (0.56–2.39)	10 (8–11)	11 (9–12)	1.8 (0.84–2.67)	10 (8–11)	10 (8–12)	0.4 (−0.48–1.06)	1.2 (−0.01–2.38)	1.5 (0.27–2.66)
4MGS^#^ (m·s^−1^)	0.92 (0.84–1.01)	0.98 (0.9–1.07)	0.11 (0.037–0.18)	1.04 (0.95–1.12)	1.06 (0.99–1.12)	0.33 (0.04–0.11)	1.03 (0.96–1.09)	1.04 (0.98–1.10)	0.01 (0.07–0.05)	0.12 (0.02–0.21)	0.04 (−0.05–0.14)
**Handgrip**^#^ **(kg)**	27.64 (23.67–31.62)	31.76 (28.1–35.43)	3.91 (2.29–5.52)	31.96 (28.98–34.95)	34.11 (30.84–37.38)	1.23 (−0.35–2.83)	31.75 (28.65–34.85)	34.21 (31–37.41)	1.85 (0.43–3.28)	2.06 (0.07–4.18)	−0.62 (−2.72–1.50)
**QMVC**^#^ **(kg)**	29.14 (24.4–33.89)	30.75 (25.78–35.72)	3.22 (0.22–6.23)	31.35 (27.69–35.01)	36.01 (32.42–39.59)	3.24 (0.31–6.18)	31.77 (28.09–35.44)	32.37 (28.96–35.75)	0.11 (−2.57–2.35)	3.33 (−0.55–7.10)	3.35 (0.43–7.10)
**EQ-5D-5L**											
Utility index^#^	0.58 (0.53–0.63)	0.61 (0.56–0.66)	0.03 (−0.04–0.10)	0.65 (0.6–0.69)	0.65 (0.62–0.69)	0.00 (−0.07–0.07)	0.59 (0.52–0.66)	0.64 (0.59–0.69)	0.05 (−0.01–0.11)	−0.02 (−0.11–0.07)	−0.05 (−0.14–0.04)
Thermo-meter^#^	57.75 (52.44–63.05)	62.4 (57.14–67.65)	4.48 (−0.42–9.38)	59.7 (54.20–65.20)	68.37 (16.70)	4.59 (−0.27–9.46)	60.84 (55.59–66.09)	65.61 (60.75–70.48)	5.56 (1.09–10.36)	−1.08 (−7.65–5.46)	0.97 (−7.38–5.73)
**PHQ-9**	9.73 (8.13–11.34)	7.78 (6.19–9.37)	1.88 (−2.99–−0.77)	8.97 (7.47–10.47)	4.50 (4.87–7.13)	1.77 (−2.91–−0.63)	10.29 (8.58–12.00)	8 (6.48–9.52)	2.29 (−3.33–−1.27)	−0.41 (−1.08–1.91)	−0.52 (−1.02–2.02)
**GAD-7**											
Severity score	7.54 (6.02–9.05)	6.27 (4.76–7.78)	1.37 (−2.51–−0.24)	7.11 (5.65–8.58)	4.81 (3.67–5.95)	−1.33 (−2.51–−0.15)	6.5 (5.04–7.96)	6.00 (4.54–7.46)	−0.81 (−1.86–0.22)	−0.56 (−2.07–0.96)	−0.52 (−2.09–1.02)
**MoCA^#^**	24.19 (23.18–25.19)	24.30 (23.19–25.40)	0.41 (−0.32–1.14)	25.16 (24.28–26.03)	26.6 (26.09–27.10)	1.07 (0.32–1.82)	24.77 (23.85–25.69)	25.05 (24.02–26.09)	0.26 (−0.39–0.91)	0.15 (−0.82–1.10)	0.81 (−0.16–1.79)
**FACIT-FS^#^**	26.23 (22.77–29.69)	32.27 (29.22–25.31)	6.36 (3.95–8.79)	29.71 (26.60–32.81)	33.73 (30.58–36.87)	1.68 (−0.78–4.15)	27.44 (24.04–30.84)	30.67 (27.50–33.84)	3.43 (1.24–5.64)	2.93 (−0.31–6.16)	−1.75 (−4.97–1.57)
**Dyspnoea-12**	11.35 (9.02–13.67)	8.60 (6.55–10.65)	−3.09 (−4.77–−1.42)	11.76 (9.28–14.24)	6.64 (4.97–8.32)	−2.66 (−4.39–0.94)	10.15 (7.78–12.52)	8.79 (6.67–10.91)	−0.98 (−2.50–0.54)	−2.11 (−4.33–0.13)	1.68 (−4.00–0.55)
**DSQ**											
Frequency	37.92 (30.22–45.63)	37.56 (28.74–46.38)	0.04 (−6.15–6.24)	38.71 (31.18–46.24)	28.49 (21.44–35.53)	−6.73 (−12.76–−0.71)	39.00 (31.35–46.65)	37.23 (30.08–44.38)	−1.01 (−6.33–4.31)	1.05 (−7.00–7.11)	−5.72 (−13.73–2.13)
Severity	32.36 (25.63–39.08)	32.38 (24.59–40.16)	−0.02 (−1.31–1.27)	32.42 (25.87–38.96)	26.40 (19.63–33.16)	−0.84 (−2.09–0.41)	36.67 (29–44.33)	35.18 (28.02–42.33)	−0.24 (−1.33–0.85)	0.22 (−1.43–1.88)	−0.6 (−2.24–1.02)

Data are presented as n or mean (95% confidence interval), unless otherwise stated. SPPB: Short Physical Performance Battery; IQR: interquartile range; 4MGS: 4-m gait speed test; QMVC: quadriceps maximal voluntary contraction; EQ-5D-5L: EuroQol 5-domain 5-level; PHQ-9: Patient Health Questionnaire 9; GAD-7: Generalised Anxiety and Depression 7; MoCA: Montreal Cognitive Assessment; FACIT-FS: Functional Assessment of Chronic Illness Therapy Fatigue Scale, DSQ: DePaul Symptom Questionnaire. ^#^: outcomes for which a higher score is an improvement.

Example flow cytometry dot plots for lymphocyte immunotyping are as shown in supplementary figure S1 (n=31). There was no significant group×time point interaction for total lymphocytes. However, significant interactions were found for central memory CD4^+^ T-cell counts, total CD8^+^ T-cell counts, naïve, central and effector memory CD8^+^ T-cell counts, and NK cell counts. These cell counts increased from pre- to post-intervention in the exercise group, but decreased in the control group. There were no other significant group×time point interactions (table 3, supplementary figure S5).

**TABLE 3:** The effect of exercise intervention *versus* control on immune cell subset changes from pre- to post-trial

	Face-to-face rehabilitation			Usual care			p-value for interaction
	Participants	Pre-trial	Post-trial	Participants	Pre-trial	Post-trial	
**Total lymphocytes (×10^9^ cells·L^−1^)**	13	2.03 (1.86–2.22)	2.22 (2.03–2.43)	18	1.92 (1.77–2.08)	1.93 (1.78–2.09)	0.19
**CD4^+^ (cells·µL^−1^)**	8	636 (442–914)	684 (476–983)	15	568 (429–752)	394 (298–522)	0.12
**CD8^+^ (cells·µL^−1^)**	10	373 (304–441)	491 (423–560)	16	399 (343–454)	324 (266–382)	0.003
**NK cells (cells·µL^−1^)**	10	271 (198–345)	378 (298–459)	16	302 (236–367)	265 (200–331)	0.04

Data are presented as n or mean (95% confidence interval), unless otherwise stated. Analyses were adjusted for sex and the baseline value of the dependent variable and for each group.

### Safety

There were two reported serious adverse events during the study period, one of which was resolved on the same day (details redacted to protect anonymity), and the other was a reported death during the study period. All were adjudged to be unrelated to the intervention.

## Discussion

In this fully powered RCT, we demonstrated that both face-to-face and remote exercise-based rehabilitation significantly improve exercise capacity compared to usual care alone in those previously hospitalised with COVID-19. These between-group improvements exceed the established minimal clinically important difference (MCID) (35 m) [24], highlighting improvements of clinical relevance in those with post-COVID syndrome.

We deliberately assessed two different models of delivering rehabilitation, avoiding direct comparison, recognising the need for models of care delivery appropriate to different segments of the population. The value of this strategy was demonstrated by the number of participants unable to uptake either intervention (94 out of 181, 52%). Traditional comparator trials would require participants to be able to access both interventions, which would have resulted in excluding 94 participants, typically those who were working age and so unable to attend face-to-face care, or those who have low digitally literacy (often compounded by health inequalities). Therefore, this randomisation procedure offered a solution to be inclusive, providing a more representative sample, but did result in some baseline differences (notably age and time since hospitalisation) which were adjusted for in the analysis.

Consistent with our primary outcome, we found potential improvements in several other physical outcomes above the established MCID when compared to usual care [25, 26]. Despite this, we did not detect improvements in self-reported HRQoL above the usual-care group. This study did not demonstrate improvements in the EQ-5D utility index between groups. Given the broad range of symptoms of post-COVID syndrome, it is plausible that many participants meet the floor or ceiling of the symptom-specific outcome measures, and that generic HRQoL tools are insensitive, and therefore specific post-COVID syndrome HRQoL measures maybe more sensitive and are now available [27, 28]. The REGAIN trial has demonstrated improvements in quality of life, measured by Patient-Reported Outcomes Measurement Information System Preference score, through a synchronised remote intervention in the absence of a measure of exercise capacity/physical function [17].

Immune dysregulation is common in post-COVID syndrome, characterised by persistent decreases in T-cell and NK immune cell populations, central to viral defence [11, 29]. Research has demonstrated reduced frequency and number of naïve CD4^+^ and CD8^+^ T-cells, increased frequency and number of memory CD4^+^ and CD8^+^ T-cells and higher frequency and absolute numbers of senescent CD4^+^ and CD8^+^ T-cells in those with severe symptoms compared to healthy controls [11]. While changes in senescent phenotypes were unchanged, numbers of naïve and memory CD4^+^ and CD8^+^ T-cell subsets increased in this study, which adds to the increasing body of evidence that exercise-based rehabilitation promotes restoration of some antiviral aspects of post-COVID syndrome-related immune dysfunction, potentially protecting against new infections. This was a substudy within the RCT and therefore has a small sample size. Further exploration of immune dysregulation and recovery through rehabilitation would be valuable. It is encouraging that there is no signal of a negative influence on the immune system by an exercise-based rehabilitation programme.

Some patients with post-COVID syndrome experience PEM/PESE, which can present a challenge. We excluded those under active investigation for, or a diagnosis of, severe and debilitating PEM (n=1) resulting in inability to leave the house, as this intervention was not deemed appropriate for this patient group. We used extensive PESE monitoring and screening in those included in the trial to ensure that symptoms were not worsened by the intervention in any participants [6]. Measures of fatigue and symptoms improved across all three groups, suggesting that the rehabilitation exercise was not harmful in appropriately selected patients The presence of severe PEM/PESE is probably higher in patients under the care of COVID clinics than reported in this trial, which is potentially the result of careful participant selection and identification.

The trial has limitations in that it included only hospitalised patients with COVID-19. Participants were randomised based on their ability to access each intervention, this led to some differences between groups with those unable to attend remote programmes being older, but we controlled for these variables in our predefined analysis plan, and this was not considered a fault of the randomisation. We found improvement in the usual-care group, particularly in relation to the EQ-5D utility index, with an increase in the summary score greater than that which has been reported previously [4]. The EQ-5D utility index is determined by five domains, which includes anxiety and depression. It is therefore possible that these improvements are related to improvements in anxiety and depression supported by similar changes in the PHQ-9.

Our population included 55% males, comparable to the overall hospitalised populations who were at higher risk of severe COVID-19 [30]. This is consistent with the gender split reported in the REGAIN trial, whereby participants were 45% males [17, 30]. While females are more at risk of post-COVID syndrome, post-hospitalised populations are biased towards males [16]*.* The age of our population is comparable to that described in the REGAIN trial. The population appears to be reflective of those who require rehabilitation, and this may be a result of those of younger age requiring less support and therefore not being referred. The results of this trial demonstrate that two modes of delivery are efficacious at improving exercise capacity in those with post-COVID syndrome following hospitalisation. Therefore, it is recommended that adults, post-hospitalisation from COVID-19 with ongoing symptoms, and functional/exercise impairment should be referred to an appropriate rehabilitation programme, with symptom-titrated exercise prescribed to individual patient needs.

Our study demonstrates the efficacy of exercise-based rehabilitation programmes when provided by two delivery methods, for adults living with post-COVID syndrome following a hospitalisation. Improving exercise capacity may have important secondary beneficial effects on the immune system in post-COVID syndrome.

## Supplementary material

10.1183/13993003.02152-2024.Supp1 **Please note:** supplementary material is not edited by the Editorial Office, and is uploaded as it has been supplied by the author.
Supplementary material ERJ-02152-2024.Supplement

## Shareable PDF

10.1183/13993003.02152-2024.Shareable1 This PDF extract can be shared freely online.
Shareable PDF ERJ-02152-2024.Shareable

## Data Availability

Data can be shared with scientists upon reasonable request to the corresponding author, ensuring relevant research training evidence is provided (*i.e*. GCP, IG).
