# Supplementary material for: Post-Hospitalisation COVID-19 Rehabilitation (PHOSP-R): a randomised controlled trial of exercise-based rehabilitation
Source: Eur Respir J. 2025 May 22;65(5):2402152. doi: 10.1183/13993003.02152-2024 (PMC12095904; doi:10.1183/13993003.02152-2024)

Online supplement

Educational topics included in the face-to-face rehabilitation programme are listed below, at the time of the study, handouts were available on a public facing website (www.yourcovidrecovery.nhs.uk). Education was delivered in a group setting, with facilitated discussion. This was delivered on a rolling programme.

Getting moving again	Fear & Anxiety	Eating well	Headaches
Breathlessness	Mood and coping	Sleeping well	Post-exertional symptom exacerbation
Cough	Memory/concentration	Managing ADLs	Next steps - active lifestyle
Fatigue	Goal setting	Return to work	Q&A

Table S1 Educational topics for face-to-face rehabilitation.

Immune biomarkers methods

Prior to and following the intervention period, venous blood samples were collected via venepuncture into EDTA- and sodium heparin-coated monovettes. Heparinised blood (12ml) was used for flow cytometric determination of immune cell subsets. EDTA blood was used for analysis of total lymphocyte counts with an automated haematology analyser.

Fluorescently conjugated antibodies were used to identify the following subsets: T cells ($CD3^+$ $CD4/CD8^+$) – naïve ($CD27^+$ $CD45RA^+$), central memory ($CD27^+$ $CD45RA^-$), effector memory ($CD27^-$ $CD45RA^-$), and terminally differentiated effector memory ($CD27^-$ $CD45RA^+$); NK cells ($CD3^-$ $CD56^+$) – (Figure 1). Fluorescence minus one controls were used to gate the aforementioned subsets. The proportions of the different subsets were used with total lymphocyte count (obtained from the haematology analyser) to calculate the circulating numbers for each subset.

To prepare samples for flow cytometry, heparinised blood was mixed with the appropriate antibodies and incubated for a total of 20min at room temperature, with BD FACS^{TM} lysing solution added at the 10-minute mark. The samples were then centrifuged at 3500rpm for 6min at 4°C. The resulting pellet from each sample was resuspended in wash buffer (Dulbecco's phosphate buffered saline (D-PBS) supplemented with 0.5% bovine serum albumin and 2 mM EDTA), before undergoing another centrifuge spin with the same configuration. Lastly, the pellet from each sample was resuspended in D-PBS before data acquisition using a 4-colour flow cytometer (Accuri C6, BD, Oxford, UK).

Figure S1 *Example gating protocol for T cells and NK cells. T cells:* ***1A*** *Selection of singlets;* ***1B*** *Selection of lymphocytes;* ***1C*** *Selection of T cells;* ***1D****; Identification of T cell subsets (Naïve, CM, EM,* T_{EMRA}*).NK cells:* ***2A*** *Selection of singlets;* ***2B*** *Selection of lymphocytes;* ***2C*** *Selection of NK cells SSC, side scatter; FSC, forward scatter; -H suffix denotes height; -A suffix denotes area; CEM, central memory; EM, effector memory;* T_{EMRA}*, terminally differentiated.*

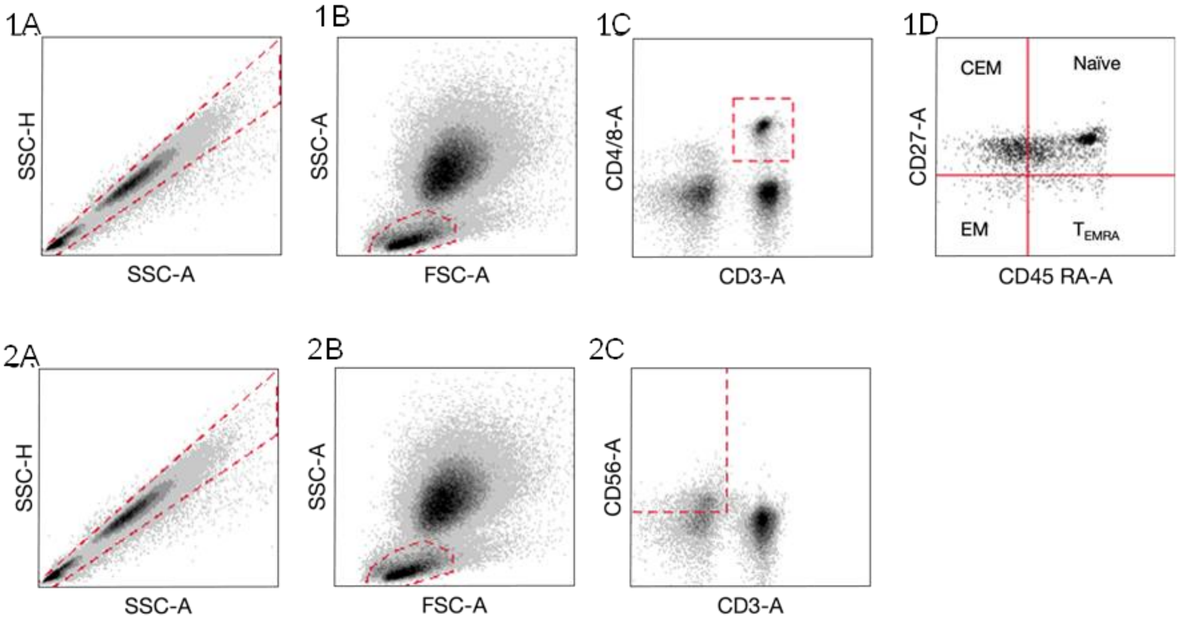

Secondary outcomes

The per protocol analysis is available in table S2. 40 participants were eligible for the face-to-face rehabilitation group, meeting 75% of class sessions attended (12/16) and attended a follow up appointment. 38 participants were eligible for the remote rehabilitation group, reaching at last phase three of four on the website platform and attending a follow up appointment.

Statistical analysis

The GLMM allows for adjustments of random effects (within-subject variability), baseline differences and accounts for missing data, assuming missing at random, providing a more accurate calculation of effects compared to other models. The final model reported the difference between and within groups. Appropriate assumptions were checked, including linearity assumption distribution and homoscedasticity of the residuals, distribution of the random effects and multicollinearity. A pre-planned additional analysis was conducted without adjusting for independent variables. A per-protocol analysis was performed on individuals with complete data on the primary outcome (attended baseline and follow-up assessment) and adherence to the intervention, defined as attending ≥75% of face-to-face sessions (12/16) or reaching phase three of the remote intervention. Data were presented as mean [95% Confidence Interval (CI), calculated using function confint method in R] unless stated otherwise. Adverse events were reported for each group and trial uptake and intervention compliance were reported as frequency data.

Linear mixed models were employed to assess the changes in immune cell counts across the study duration among different groups. Fixed effects in the linear mixed models included: group (exercise intervention vs control), time point (pre vs post), group*time, the baseline value of the dependent variable and any potential confounders with significant differences between groups at baseline. The participant identifier was treated as a random effect.

Assumptions of GLMM

The assumptions assessed for the GLMM with Gaussian function link are:

- Linear behavior of the covariates and the target variable (scatter plot)
- Residuals follow a normal distribution around the zero (QQ plot and Shapiro-Wilk test)
- Random effects follow a normal distribution (Q-Q plot)
- Homoscedasticity (Levene's Test)
- Collinearity

ITT Model

Linearity covariates and output

Figure S2. Residuals plot of the model showing that residuals are randomly distributed around zero, linearity assumption check.

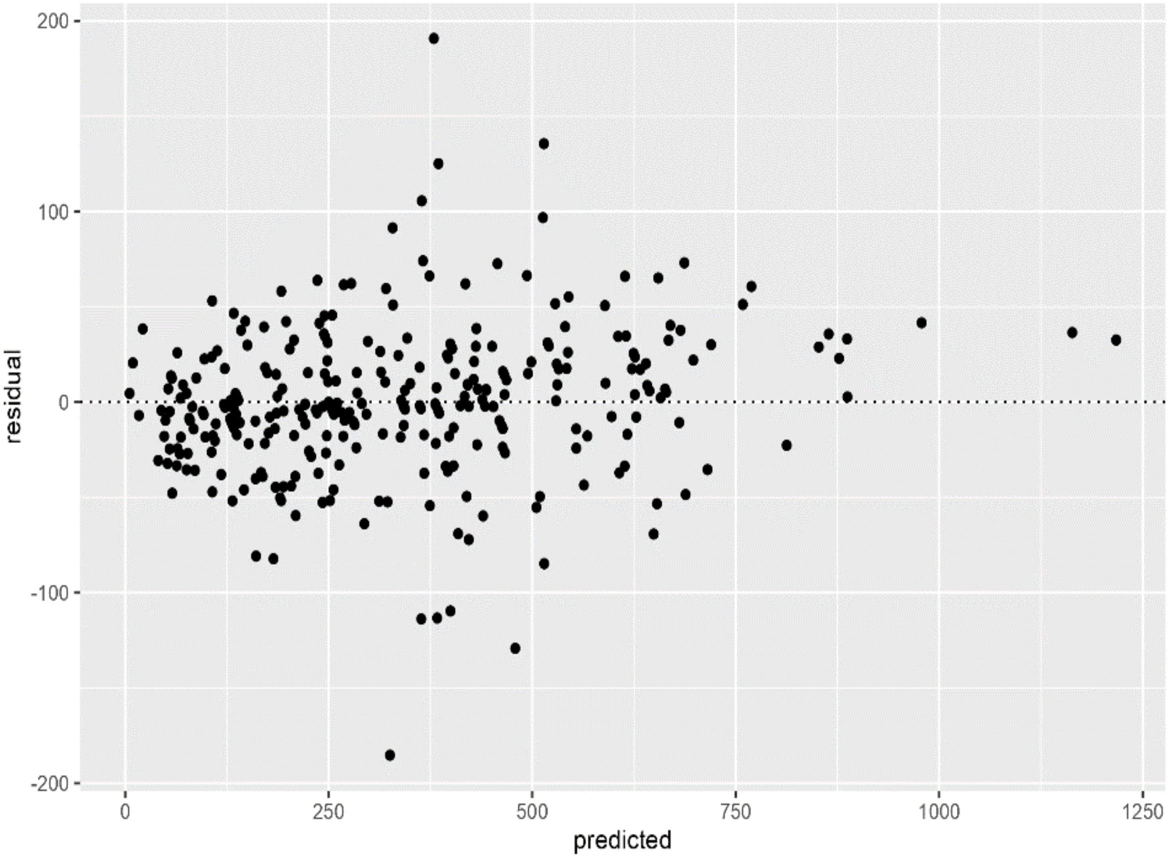

Normality of the residuals

Figure S3. Q-Q Plot of the ITT model assessing the assumption of distribution of normality of the residuals with Shapiro-Wilk test

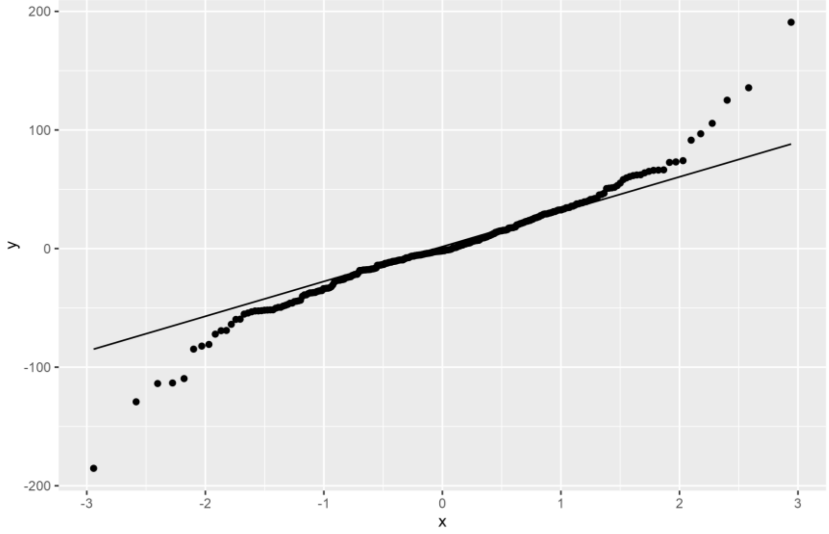

Shapiro-Wilk Test: W = 0.95389, p-value = 3.028e-08

Normality of Random Effects

Figure S4 Q-Q Plot of the ITT model assessing the assumption of distribution of normality of the random effects with Shapiro-Wilk test

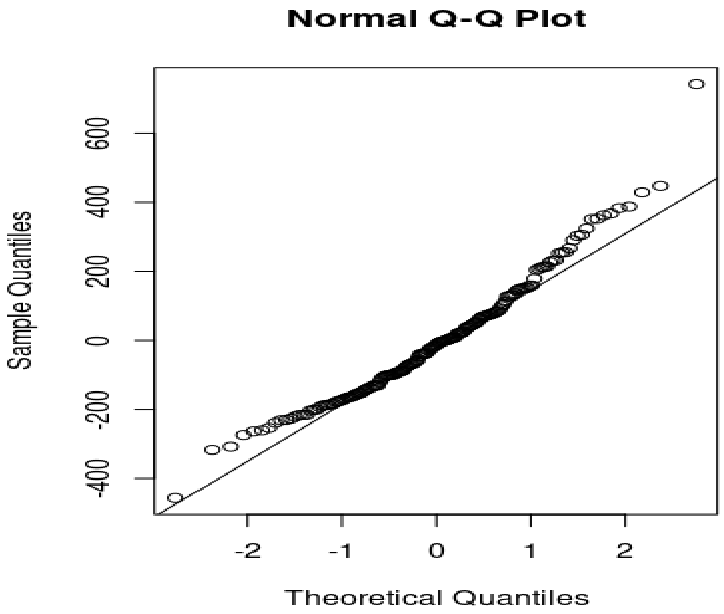

Shapiro-Wil Test : W = 0.96722, p-value = 0.0004828

Homoscedasticity (homogeneity of the variance)

Table S2 Levene's Test to test Homogeneity of Variance

	Df	F Value	P-Value
Group	5	0.0635	0.9973

Multicollinearity

Covariate	VIF [95%IC]
age_treatment	1.24 [1.13 – 1.46]
crf1a_Sex	1.72 [1.51 – 2.04]
BMI	1.76 [1.54 – 2.08]
Time Since Hosp	1.09 [1.02 – 1.57]
Resp_support_3levels	1.33 [1.20 – 1.57]
Site	1.38 [1.23 – 1.62]
Period_point	2.55 [2.17 – 3/06]
treatment_group	1.33 [1.19 – 1.56]

	Face-to-face n=40			Remote n=38			Usual care n=62			Face-to-face vs Usual care	Remote vs Usual care
	Pre-	Post-	Change	Pre-	Post-	Change	Pre-	Post-	Change	Difference	Difference
ISWT	252[179 to 324]	319[238 to 400]	67[41 to 94]	363[289 to 437]	409[341 to 476]	44[15 to 73]	328[277 to 380]	331[280 to 381]	2[-20 to 23]	66[32 to 99]	42[7 to 78]
SPPB	9(7 to 11)	11(9 to 12)	1.47(0.56 to 2.39)	10(9 to 11)	11(10 to 12)	1.75(0.84 to 2.67)	10(8 to 11)	10(8 to 12)	0.29(-0.47 to 1.06)	1.18(-0.01 to 2.38)	1.46(0.27 to 2.66)
4MGS	0.88[0.79 to 0.96]	1.01[0.91 to 1.11]	0.14[-0.06 to 0.22]	1.08[0.98 to 1.18]	1.09[1 to 1.18]	0.01[-0.07 to 0.1]	1.03[0.96 to 1.09]	1.04[0.98 to 1.1]	-.01[-0.07 to 0.05]	0.15[0.05 to 0.24]	-0.02[-0.08 to 0.12]
Handgrip	26.92[22.18 to 31.67]	31.93[27.54 to 36.31]	3.93[2.11 to 5.76]	33.52[29.88 to 37.15]	36.72[32.75 to 40.69]	1.63[-0.33 to 3.60]	31.75[28.65 to 34.85]	24.21[31.00 to 37.41]	1.83[0.36 to 3.31]	2.10[-0.196 to 4.42]	-0.20[-2.59 to 2.24]
QMVC	27.33[21.57 to 33.10]	31.79[25.95 to 37.62]	4.16[0.89 to 7.43]	31.67[27.03 to 36.91]	36.15[31.09 to 41.20]	3.64[0.16 to 7.12]	31.77[28.09 to 35.44]	32.37[28.96 to 35.78]	-0.12[-2.63 to 2.38]	4.28[0.22 to 8.30]	3.76[-0.46 to 7.96]
EQ5D 5L											
Utility Index	0.60[0.54 to 0.66]	0.62[0.56 to 0.68]	0.02[-0.06 to 0.10]	0.66[0.61 to 0.72]	0.67[0.61 to 0.72]	0.00[-0.09 to 0.08]	0.59[0.52 to 0.66]	0.64[0.59 to 0.69]	0.04 [-0.02 to 0.011]	-0.02 [-0.13 to 0.08]	-0.04 [-0.15 to 0.05]
Thermometer	56.95[50.86 to 63.04]	62.84[56.87 to 68.81]	4.96 [-0.70 to 10.62]	63.53[53.89 to 71.17]	67.52[61.53 to 73.50]	1.96[-4.16 to 8.09]	60.84 [55.59 to 66.09]	65.61 [60.75 to 70.48]	5.58[0.93 to 10.24]	-0.62[-7.87 to 6.58]	-3.62 [-11.07 to 4.13]
PHQ9	9.63[7.64 to 11.61]	7.50[5.63 to 9.37]	-1.78[-2.98 to -0.59	6.95[5.23 to 8.66]	5.1[3.82 to 6.38]	-1.44[-2.76 to 0.12]	10.29 [8.58 to 12.00]	8.00[6.48 to 9.52]	-2.29[-3.31 to 1.29]	0.51[-1.04 to 2.04]	0.85[-0.79 to 2.48]
GAD7											
Severity score	7.40[5.59 to 9.21]	6.08 [4.24 to 7.91]	-1.07 [-2.29 to 0.15]	5.38[3.85 to 6.91]	3.93[2.72 to 5.15]	-1.38[-2.75 to 0.02]	6.50[5.04 to 7.96]	6.00[4.54 to 7.46]	-0.82[-1.84 to 0.19]	-0.25[-1.81 to 1.31]	-0.56[-2.23 to 1.11]
Inference score	2 [2 to 3]	2 [1 to 2]	-0.44 [-1.25 to 0.46]	2 [2 to 2]	2 [1 to 2]	-0.59 [-1.56 to 0.38]	2 [1 to 3]	12 [2 to 3]	-.13 [-0.66 to 0.86]	-0.57 [-1.74 to 0.6]	-0.72 [-1.94 to 0.5]
MoCA	MoCA	24.05[22.87 to 25.23]	24.00[22.68 to 25.32]	0.06[-0.74 to 0.86]	25.71[24.84 to 26.58]	27.07[26.47 to 27.68]	1.02[0.135 to 1.92]	24.77[23.85 to 25.69]	25.05[24.02 to 26.09]	0.26[-0.38 to 0.90]	-0.20[-1.21 to 0.80]
FACIT-FS	FACIT-FS	26.43[22.26 to 30.59]	32.86[29.31 to 36.48]	6.06[3.34 to 8.78]	33.09[29.36 to 36.82]	35.23[31.24 to 39.21]	1.21[-1.74 to 4.17]	27.44[24.04 to 30.84]	30.67[27.50 to 33.84]	3.44[1.21 to 5.68]	2.62[-0.85 to 6.09]
Dyspnoea-12	Dyspnoea-12	10.85[8.11 to 13.58]	8.08[5.68 to 10.48]	-2.68[-4.40 to -0.96]	9.68[6.95 to 12.42]	6.52[4.44 to 8.60]	-1.73[-3.64 to 0.18]	10.15[7.78 to 12.52]	8.79[6.67 to 10.91]	-0.97[-2.38 to 0.44]	-1.71[-3.89 to 0.49]
DSQ											
Frequency	39.05[30.03 to 48.08]	34.71[24.49 to 44.92]	-1.71 [-16.17 to -2.01]	35.00[25.26 to 44.74]	24.50[15.39 to 33.61]	-9.08 [-6.28 to 4.23]	39.00[31.35 to 46.65]	37.23[30.08 to 44.38]	-1.02[-8.57 to 5.15]	-0.69[-9.21 to 7.79]	-8.06 [-16.77 to 0.56]
Severity	32.97[25.3 to 40.65]	29.09[20.41 to 37.77]	-0.36[-1.83 to 1.09]	30.41[21.21 to 39.60]	21.00[12.67 to 29.33]	-1.70[-3.18 to -0.22]	36.67[29.00 to 44.33]	35.18[28.02 to 42.33]	-0.24[-1.34 to 0.85]	-0.12[-1.92 to 1.66]	-1.46[-3.28 to 0.34]

Table S3 Per protocol analysis for all outcomes for face-to-face rehabilitation vs usual care, and remote rehabilitation vs usual care presented as mean[95% CI] or median (25th – 75th quartile). *SPPB Short Physical Performance Battery, QMVC Quadriceps Maximal Voluntary Contraction, EQ5D-5L EuroQol 5*

Domain- 5 Level, PHQ9 Patient Health Questionnaire 9, GAD7 Generalised Anxiety and Depression 7, MoCA Montreal Cognitive Assessment FACIT-FS Functional Assessment of Chronic Illness Therapy Fatigue Scale, DSQ DePauls Symptom Questionnaire.

Brief pain inventory

The Brief Pain Inventory is completed in full if participants indicate they are experiencing any pain. Therefore there are data available on 118 participants for the primary analysis and 92 participants for the per protocol analysis. The results of the Brief Pain Inventory is presented in table SX.

	Face-to-face n=36			Remote n=42			Usual Care n=40			Face-to-face vs Usual care	Remote vs Usual care
	Pre-	Post-	Change	Pre-	Post-	Change	Pre-	Post-	Change	Difference	Difference
BPI severity	16.44 [14.07 to 18.81]	16.37 [13.57 to 19.17]	1.34 [-2.12 to 4.81]	12.25 [10.17 to 14.33]	11.09 [8.64 to 13.54]	-1.99 [-5.89 to 1.91]	13.90[11.82 to 15.97]	13.48[11.34 to 15.63]	0.23[-2.84 to 3.31]	1.11 [-3.57 to 5.49]	-2.22 [-0.71 to 2.58]
BPI interference	36.22 [30.43 to 42.01]	23.14 [17.88 to 28.40]	-8.91 [-14.89 to -2.94]	26.17 [21.14 to 31.19]	22.42 [18.26 to 26.59]	-5.1 [-11.15 to 0.94]	27.40[22.63 to 32.17]	24.43[19.71 to 29.14]	-4.87[-10.05 to 0.30]	-4.04 [-12.03 to 3.48]	-0.23 [-8.33 to 7.33]
Per protocol analysis											
	Face-to-face n=26			Remote n=27			Usual Care n=39			Face-to-face vs Usual care	Remote vs Usual care
BPI severity	16.54[13.49 to 19.59]	17.36[13.96 to 2.77]	3.11[-0.59 to 6.81]	11.39[8.40 to 14.38]	8.86[5.67 to 12.04]	-3.97[-8.74 to 0.80]	13.90[11.82 to 15.97]	13.48[11.34 to 15.63]	0.21[-2.67 to 3.10]	2.90[-1.86 to 7.26]	-4.18[-9.55 to 1.22]
BPI interference	35.81[29.15 to 42.46]	20.04[14.05 to 26.46]	-9.83[-16.70 to 2.97]	22.89[16.69 to 29.09]	20.53[14.78 to 26.28]	-6.35[-13.66 to 0.96]	27.40[22.63 to 32.17]	24.43[19.71 to 29.14]	-4.84[-10.01 to 0.33]	-4.99[-13.73 to 3.06]	-1.51[-10.26 to 7.09]

Table S4 Primary and per protocol analysis for the Brief Pain Inventory (BPI) for face-to-face rehabilitation vs usual care, and remote rehabilitation vs usual care presented as mean[95% CI].

*Figure S5 The effect of face-to-face rehabilitation (Ex) vs Usual Care (Con) on CD4+ (A-D) and CD8+ (E-H) subset changes from pre- to post-trial. Data are mean (95% CI) and adjusted for sex and baseline value of the dependent variable. * Sig difference from pre, within trial. CD4+ subsets; n=7 for Ex, N=15 for Con. CD8+ subsets; n=10 for Ex, n=16 for Con. CM, central memory; EM, Effector Memory; TEMRA, terminally differentiated effector memory.*

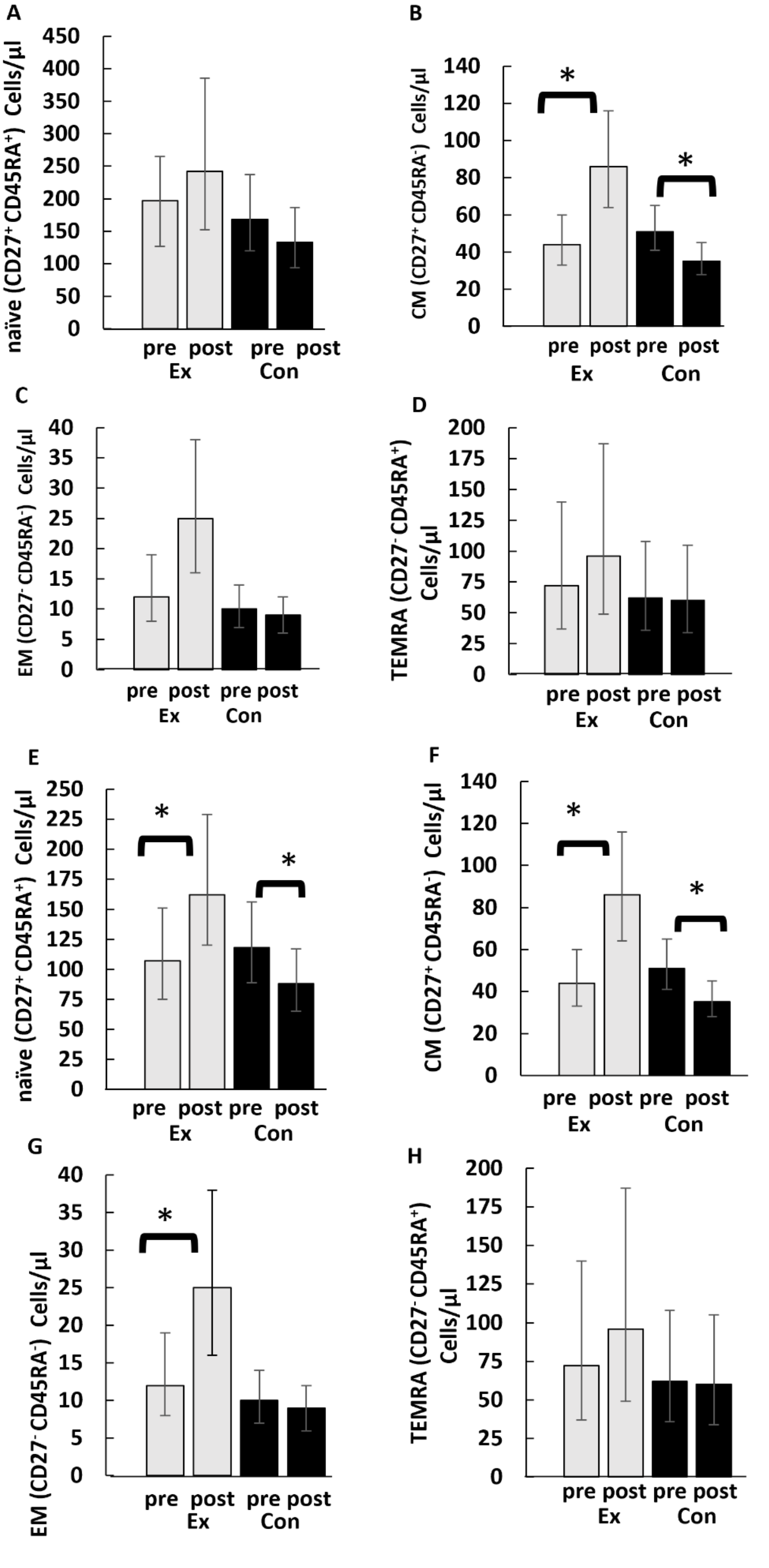

Supplement: Supplementary file 1 [file ERJ-02152-2024.Supplement.pdf]
