# Supplementary material for: Post-Hospitalisation COVID-19 Rehabilitation (PHOSP-R): a randomised controlled trial of exercise-based rehabilitation
Source: Eur Respir J. 2025 May 22;65(5):2402152. doi: 10.1183/13993003.02152-2024 (PMC12095904; doi:10.1183/13993003.02152-2024)
Supplement: Supplementary file 2 [file ERJ-02152-2024.Shareable.pdf]

SHAREABLE PDF

Post-Hospitalisation COVID-19 Rehabilitation (PHOSP-R): a randomised controlled trial of exercise-based rehabilitation

Enya Daynes, Rachael A. Evans, Neil J. Greening, Nicolette C. Bishop, Thomas Yates, Daniel Lozano-Rojas, Kimon Ntotsis, Matthew Richardson, Molly M. Baldwin, Malik Hamrouni, Emily Hume, Hamish McAuley, George Mills, Dimitrios Megaritis, Matthew Roberts, Charlotte E. Bolton, James D. Chalmers, Trudie Chalder, Annemarie B. Docherty, Omer Elneima, Ewen M. Harrison, Victoria C. Harris, Ling P. Ho, Alex Horsley, Linzy Houchen-Wolloff, Olivia C. Leavy, Michael Marks, Krishna Poinasamy, Jennifer K. Quint, Betty Raman, Ruth M. Saunders, Aarti Shikotra, Amisha Singapuri, Marco Sereno, Sarah Terry, Louise V. Wain, William D-C. Man, Carlos Echevarria, Ioannis Vogiatzis, Christopher Brightling and Sally J. Singh, on behalf of the PHOSP-COVID Study Collaborative Group

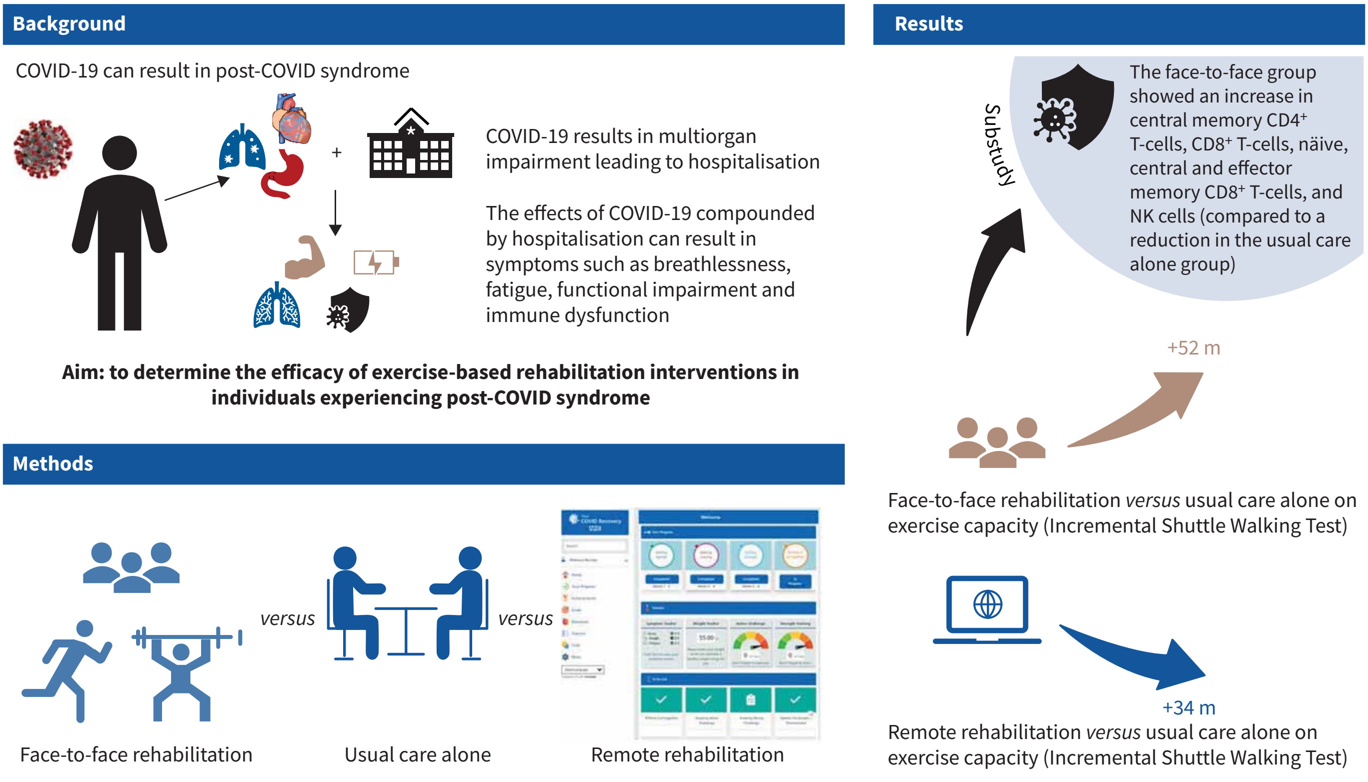

GRAPHICAL ABSTRACT In this study individuals with post-COVID syndrome improved exercise capacity following face-to-face rehabilitation or digital rehabilitation compared to usual care alone.

EUROPEAN RESPIRATORY JOURNAL
ORIGINAL RESEARCH ARTICLE
E. DAYNES ET AL.

SHAREABLE PDF

Post-Hospitalisation COVID-19 Rehabilitation (PHOSP-R): a randomised controlled trial of exercise-based rehabilitation

Enya Daynes [1,2], Rachael A. Evans [1,2], Neil J. Greening [1,2], Nicolette C. Bishop[3], Thomas Yates[4,5], Daniel Lozano-Rojas[1,2], Kimon Ntotsis[1,2], Matthew Richardson[1,2], Molly M. Baldwin [1,2], Malik Hamrouni[5], Emily Hume [6], Hamish McAuley [1,2], George Mills[1,2], Dimitrios Megaritis [6], Matthew Roberts[5], Charlotte E. Bolton [7,8], James D. Chalmers[9], Trudie Chalder[10], Annemarie B. Docherty[11], Omer Elneima [1,2], Ewen M. Harrison[12], Victoria C. Harris[1], Ling P. Ho[12,13], Alex Horsley [14], Linzy Houchen-Wolloff [1,2], Olivia C. Leavy[1,15], Michael Marks [16,17], Krishna Poinasamy[18], Jennifer K. Quint [19], Betty Raman [20,21], Ruth M. Saunders[1], Aarti Shikotra[1], Amisha Singapuri[1], Marco Sereno[1], Sarah Terry[1], Louise V. Wain [1,15], William D-C. Man [22,23,24], Carlos Echevarria[25], Ioannis Vogiatzis[6], Christopher Brightling[1,2] and Sally J. Singh [1,2], on behalf of the PHOSP-COVID Study Collaborative Group

[1]The Institute for Lung Health, NIHR Leicester Biomedical Research Centre, University Hospitals of Leicester, Leicester, UK. [2]Department of Respiratory Sciences, University of Leicester, Leicester, UK. [3]National Centre for Sport and Exercise Medicine, School of Sport, Exercise and Health Sciences, Loughborough University, Loughborough, UK. [4]NIHR Leicester Biomedical Research Centre – Diabetes, Leicester, UK. [5]Diabetes Research Centre, College of Life Sciences, University of Leicester, Leicester, UK. [6]Department of Sport, Exercise and Rehabilitation, Faculty of Health and Life Sciences, Northumbria University, Newcastle upon Tyne, UK. [7]Centre for Respiratory Research, Translational Medical Sciences, School of Medicine, University of Nottingham, Nottingham, UK. [8]NIHR Nottingham Biomedical Research Centre, Nottingham, UK. [9]University of Dundee, Ninewells Hospital and Medical School, Dundee, UK. [10]Institute of Psychiatry, Psychology and Neuroscience, King's College London, London, UK. [11]Centre for Medical Informatics, The Usher Institute, University of Edinburgh, Edinburgh, UK. [12]MRC Human Immunology Unit, University of Oxford, Oxford, UK. [13]NIHR Oxford Biomedical Research Centre, Oxford, UK. [14]Manchester University NHS Foundation Trust, Manchester, UK. [15]Department of Population Health Sciences, University of Leicester, Leicester, UK. [16]Department of Clinical Research, London School of Hygiene and Tropical Medicine, London, UK. [17]Hospital for Tropical Diseases, University College London Hospital, London, UK. [18]Asthma and Lung UK, London, UK. [19]School of Public Health, Imperial College London, London, UK. [20]Radcliffe Department of Medicine, University of Oxford, Oxford, UK. [21]Oxford University Hospitals NHS Foundation Trust, Oxford, UK. [22]Harefield Respiratory Research Group, Royal Brompton and Harefield Hospitals, Guy's and St Thomas' NHS Foundation Trust, London, UK. [23]National Heart and Lung Institute, Imperial College, London, UK. [24]King's Centre for Lung Health, Faculty of Life Sciences and Medicine, King's College London, London, UK. [25]The Newcastle upon Tyne Hospitals NHS Foundation Trust, Translational and Clinical Research Institute, Newcastle University, Newcastle upon Tyne, UK.

Corresponding author: Enya Daynes (enya.daynes@uhl-tr.nhs.uk)

Check for updates

Shareable abstract (@ERSpublications)
Rehabilitation improved short-term exercise capacity in long COVID replicated by real-world evidence and had beneficial immunomodulatory effects https://bit.ly/42l0wzk

Cite this article as: Daynes E, Evans RA, Greening NJ, *et al*. Post-Hospitalisation COVID-19 Rehabilitation (PHOSP-R): a randomised controlled trial of exercise-based rehabilitation. *Eur Respir J* 2025; 65: 2402152 [DOI: 10.1183/13993003.02152-2024].

This PDF extract can be shared freely online.

Copyright ©The authors 2025.

This version is distributed under the terms of the Creative Commons Attribution Licence 4.0.

This article has an editorial commentary:

Abstract

Objective Post-COVID syndrome involves prolonged symptoms with multisystem and functional impairment lasting ⩾12 weeks after acute coronavirus disease 2019 (COVID-19). We aimed to determine the efficacy of exercise-based rehabilitation interventions, either face-to-face or remote, compared to usual care in individuals experiencing post-COVID syndrome following a hospitalisation with acute COVID-19.

Design This single-blind randomised controlled trial compared two exercise-based rehabilitation interventions (face-to-face or remote) to usual care in participants with post-COVID syndrome following a hospitalisation. The interventions were either a face-to-face or remote 8-week programme of individually

https://doi.org/10.1183/13993003.00239-2025

Received: 11 Nov 2024
Accepted: 13 Jan 2025

prescribed exercise and education. The primary outcome was the change in Incremental Shuttle Walking Test (ISWT) following 8 weeks of intervention (either face-to-face or remote) compared to usual care. Other secondary outcomes were measured including health-related quality of life (HRQoL), and exploratory outcomes included lymphocyte immunotyping.

Results 181 participants (55% male, mean±SD age 59±12 years, length of hospital stay 12±19 days) were randomised. There was an improvement in the ISWT distance following face-to-face rehabilitation (mean 52 m, 95% CI 19–85 m; p=0.002) and remote rehabilitation (mean 34 m, 95% CI 1–66 m; p=0.047) compared to usual care alone. There were no differences between groups for HRQoL self-reported symptoms. Analysis of immune markers revealed significant increases in naïve and memory $CD8^+$ T-cells following face-to-face rehabilitation *versus* usual care alone (p<0.001, n=31).

Conclusion Exercise-based rehabilitation improved short-term exercise capacity in post-COVID syndrome following an acute hospitalisation and showed potential for beneficial immunomodulatory effects.
